# Elevated expression of immune checkpoints and pro-inflammatory cytokines as potential biomarkers in pediatric Vulvar Lichen Sclerosus

**DOI:** 10.1038/s41598-025-33630-2

**Published:** 2026-02-02

**Authors:** Ewelina Grywalska, Paulina Mertowska, Sebastian Mertowski, Monika Zaborek-Łyczba, Jakub Łyczba, Ewa Woźniakowska, Karolina Rasoul-Pelińska, Kamila Ćwik, Anna Torres

**Affiliations:** 1 Department of Experimental Immunology, Medical University of Lublin, 20-093 Lublin, Poland; 2 Department of Pediatric and Adolescent Gynecology, Medical University of Lublin, 20-059 Lublin, Poland; 3 Laboratory of Management Education in Health Care, Medical University of Lublin, 20-093 Lublin, Poland

**Keywords:** Lichen Sclerosus et Atrophicus, Child, Cytokines, Immune system, Immunology, Autoimmunity, Inflammation

## Abstract

Vulvar Lichen Sclerosus (VLS) is a chronic inflammatory dermatosis of unknown etiology affecting the external genitalia. In pediatric patients, it can lead to significant discomfort and progressive structural changes in tissues. In recent years, there has been increasing interest in the participation of immune checkpoints and inflammatory mediators in the pathogenesis of chronic diseases, including VLS. Immune checkpoints, such as PD-1, PD-L1, CTLA-4, CD200, and CD200R, play a crucial role in modulating the immune response and may serve as potential diagnostic markers and therapeutic targets. This study aimed to evaluate the expression of PD-1, PD-L1, CTLA-4, CD200, and CD200R molecules on CD4+ T cells, CD8+ T cells, and CD19+ B lymphocytes in prepubertal girls diagnosed with VLS. Additionally, the concentrations of their soluble forms and the levels of proinflammatory cytokines, including IL-2, IL-6, and TNF-α, in serum were determined to assess their potential as diagnostic biomarkers. The study included patients with VLS (study group) and healthy children (control group). The expression of checkpoints was analyzed using flow cytometry, while the concentrations of soluble forms and cytokines were determined using the enzyme-linked immunosorbent assay (ELISA) technique. Statistical significance tests and Spearman’s rank correlation analysis were used. Patients with VLS showed markedly higher serum C-reactive protein (CRP) levels compared with healthy controls (49.99 ± 6.09 mg/L vs. 2.70 ± 0.96 mg/L, *p* < 0.001). A significant increase in checkpoint expression was observed on lymphocyte subsets, including CD4+ T cells expressing PD-1 (4.90 ± 1.67% vs. 0.85 ± 0.56%, *p* < 0.001) and CTLA-4 (11.58 ± 4.70% vs. 0.96 ± 0.48%, *p* < 0.001), as well as CD8+ T cells expressing PD-1 (17.31 ± 4.81% vs. 0.76 ± 0.67%, *p* < 0.001) and CD19+ B cells expressing PD-L1 (16.10 ± 9.50% vs. 1.41 ± 0.45%, *p* < 0.001). Soluble checkpoint molecules were consistently elevated, for example, sPD-1 (26.69 ± 3.88 pg/mL vs. 4.40 ± 0.75 pg/mL, *p* < 0.001) and sCTLA-4 (43.96 ± 3.77 pg/mL vs. 5.09 ± 1.09 pg/mL, *p* < 0.001). Similarly, cytokine levels were significantly increased in VLS patients, including interleukin-2 (28.80 ± 6.36 pg/mL vs. 4.32 ± 1.33 pg/mL, *p* < 0.001), interleukin-6 (25.35 ± 7.52 pg/mL vs. 2.35 ± 0.78 pg/mL, *p* < 0.001), and tumor necrosis factor alpha (31.26 ± 3.10 pg/mL vs. 12.80 ± 1.30 pg/mL, *p* < 0.001). Correlation analyses confirmed significant positive associations between cytokine concentrations and checkpoint expression, highlighting their interdependence in VLS immunopathogenesis. The obtained results confirm an increased immunoactivation profile in children with VLS, characterized by elevated checkpoint expression and increased levels of proinflammatory cytokines. The studied parameters show potential as diagnostic and prognostic biomarkers, which may constitute the basis for the development of new diagnostic tools and targeted therapeutic strategies in VLS in pediatric patients.

**Supplementary Information:**

The online version contains supplementary material available at 10.1038/s41598-025-33630-2.

## Introduction

Lichen sclerosus (LS)/Vulvar Lichen Sclerosus (VLS) is a chronic, non-infectious inflammatory dermatosis of unknown etiology, most often located in the external genitalia. This disease occurs in both sexes, but it is much more common in women, and the ratio of cases in women to men, according to various sources, ranges from 3:1 to 10:1. In the female population, LS can develop at any age. Still, it is particularly common in postmenopausal women and pre-pubertal girls. The incidence in the pediatric population is estimated at 0.5% in boys (mean age of diagnosis 9–11 years) and 0.11% in girls (mean age of diagnosis 7.6 years). The diagnostic delay in this group may be from 1.5 to 2 years^1–6^.

In adult women, VLS most often develops during the peri- and postmenopausal period, suggesting a significant role of hormonal factors, notably reduced estrogen levels, in the pathogenesis of this disease. Estrogens play a crucial role in maintaining the homeostasis of the skin and mucous membranes, regulating the synthesis of collagen and other components of the extracellular matrix, stimulating angiogenesis, and influencing wound healing processes^7,8^. Their deficiency leads to thinning of the epidermis and dermis, reduced tissue elasticity, atrophy of the lipid layer, and increased susceptibility to mechanical injury, which in turn exacerbates inflammatory processes and promotes the perpetuation of chronic inflammation^9,10^. Estrogens have also been shown to exert immunomodulatory effects by regulating the activity of both T lymphocytes and macrophages, modulating the balance between proinflammatory and tolerogenic responses^11^. Estrogen deficiency disrupts this balance, which may promote chronic activation of the immune system, the development of autoimmunity, and the development of fibrotic changes characteristic of VLS. In prepubertal girls, the etiopathogenesis of the disease likely proceeds via a different mechanism, as low estrogen levels are a physiological state during this period. In this age group, increasing attention is being paid to the importance of adrenarche hormones, primarily adrenal androgens such as dehydroepiandrosterone (DHEA) and its sulfate (DHEA-S). These hormones influence keratinocyte proliferation and differentiation, modulate the immune response, and may play a role in initiating local inflammatory processes^12–14^. It has also been suggested that, due to the immaturity of the pediatric immune system, including different proportions of lymphocyte subpopulations and immature mechanisms of inflammatory response regulation, the course of VLS may be characterized by a distinct immunological profile compared to that of adult women.

The most common symptom of VLS is itching. Other symptoms that may accompany itching or present as the sole ailment include burning, pain, constipation, or dysuria. In about 10% of cases, VLS is asymptomatic. What is essential is that the severity of symptoms may not be proportional to the degree of signs present on the anogenital area. The typical sign of VLS is whitening of the skin and mucosa, usually well delineated and often accompanied by fresh or healed fissures, erosions, hyperkeratosis, “cigarette paper” atrophic areas, and subcutaneous extravasations or hematomas. The signs often form a shape of an hourglass or “figure of eight” around the vaginal orifice and anus. They may also spread to the area of the intergluteal cleft^15–17^.

VLS is a progressive disease, and when not treated, it leads to serious complications, including scarring and irreversible anatomical changes to the vulvar architecture. These changes cause functional disability, including constant vulvar discomfort, dysuria, painful bowel movements or constipation, and problems with sexual intercourse and future childbirth^18–21^.

The etiopathogenesis of VLS remains multifactorial, including genetic, hormonal, environmental, and immunological components^17,22^. A significant role is attributed to the development of inflammation, which is primarily caused by autoimmune mechanisms, disorders in regulating the immune response, and reactions to environmental and genetic factors. Some studies suggest that patients with VLS tend to have comorbidities, such as vitiligo, autoimmune thyroid disease, celiac disease, diabetes, psoriasis, pernicious anemia, or alopecia areata^23,24^. An excess of interleukins may contribute to the chronic inflammation characteristic of VLS. Interleukins stimulate proliferation and activation of immune system cells, including T lymphocytes and macrophages, which may contribute to tissue damage and clinical symptoms of the disease. An excess of pro-inflammatory interleukins, such as IL-6, IL-1β, TNF-α, or IL-2, may be associated with continuous activation of the immune system, which can result in the stimulation of immune checkpoint expression as a defense mechanism aimed at limiting an overly strong inflammatory response and tissue damage^9,25,26^. Overexpression of immune checkpoints (PD-1, PD-L1) may be associated with the inhibition of the immune system’s response, which, on the one hand, may protect against autoimmunity, but on the other hand, may also inhibit an effective response against pathogens or altered cells. Moreover, in response to chronic inflammation, increasing the expression of checkpoints may reduce the activity of lymphocytes and other immune system cells, which, in the long term, may be related to the maintenance of chronic inflammation and disease progression. To date, studies examining immune checkpoints in VLS have been conducted almost exclusively in adults. In contrast, the immune profile in children may differ due to the different hormonal context, immaturity of the immune system, and the specific course and progression of the disease. Understanding the interplay between checkpoints and cytokines in the early phase of VLS during developmental age may provide valuable information for earlier diagnosis and the design of targeted therapeutic interventions. Considering this context, this study fills a significant cognitive gap and represents both a novel and necessary step toward a better understanding of the pathogenesis of pediatric VLS^27–32^.

Our study aimed to assess the occurrence of selected immune checkpoints with suppressor properties and their ligands (PD-1/PD-L1, CTLA-4, and CD200R/CD200) on subpopulations of CD4+ T lymphocytes, CD8+ T lymphocytes, and CD19+ B lymphocytes in newly diagnosed and untreated pediatric prepubertal subjects with VLS, as well as in healthy volunteers. The analyses were also accompanied by assessing the soluble forms of the tested molecules in the patients’ serum and the concentration of selected pro-inflammatory cytokines (IL-2, IL-6, TNF-α). The obtained results were correlated with the clinical parameters of the patients to identify the immunological mechanisms involved in the pathogenesis of this disease and to assess the potential of the tested molecules as biomarkers.

## Results

### Hematological, biochemical, and immunophenotypic profile of patients with Vulvar Lichen Sclerosus and healthy controls enrolled in the study

**Fig. 1:**
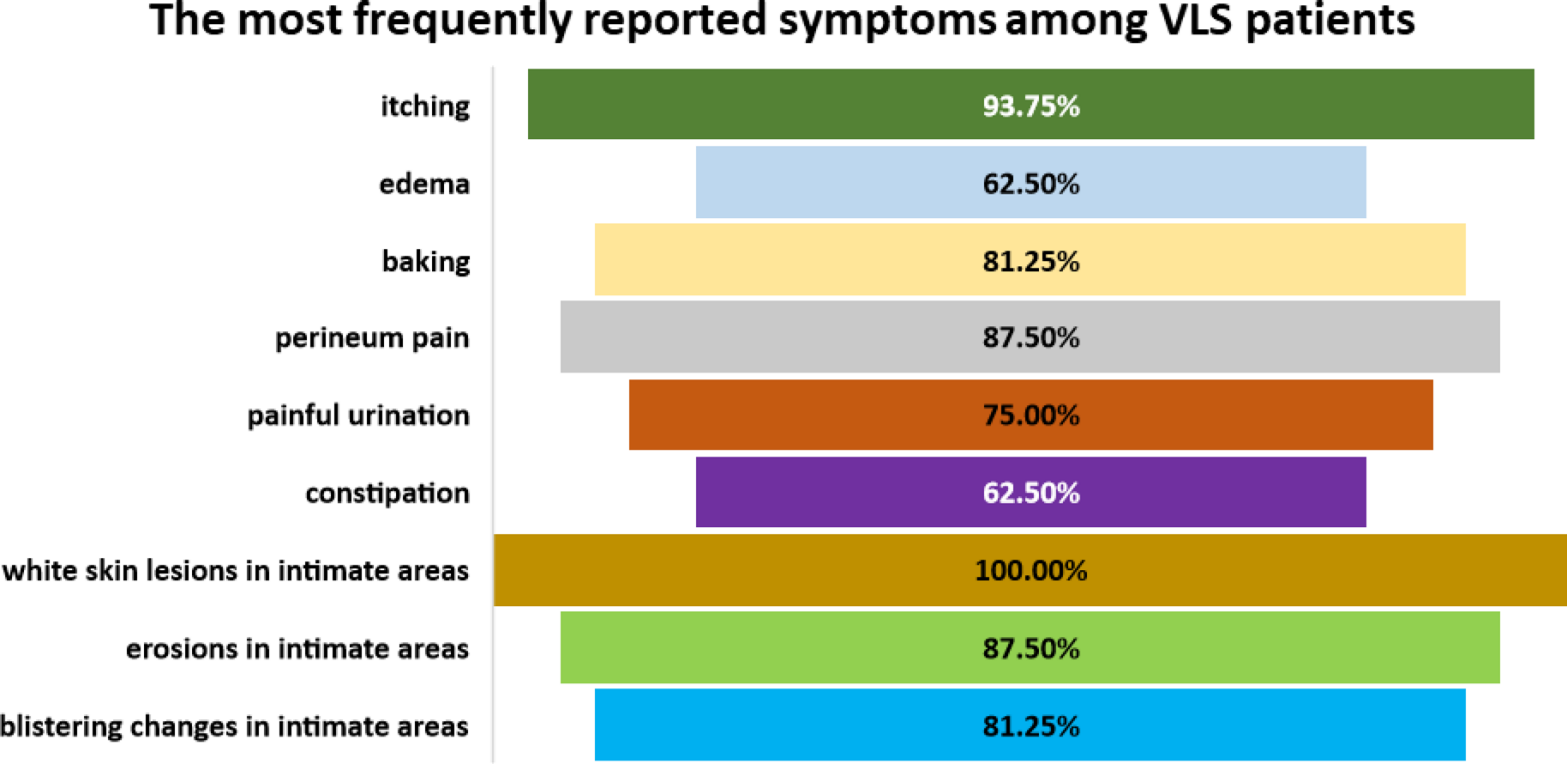
Frequency of the most commonly reported symptoms in patients with newly diagnosed Vulvar Lichen Sclerosus (VLS).

The study cohort comprised 16 treatment-naïve female patients with newly diagnosed VLS, aged between 3.75 and 17.7 years (mean age: 6.78 ± 2.15 years; median: 9 years), along with 16 age-matched healthy female controls (mean age: 7.83 ± 4.4 years; median: 6 years; age range: 4–9 years). Following detailed anamnesis and clinical examination, all VLS patients exhibited signs and symptoms characteristic of the disease (Fig. 1). Pruritus was the most prevalent complaint, reported by 75% of patients. Other reported symptoms included vulvar soreness in 25% and pain in 16.7% of cases. Defecatory and micturition difficulties were noted in 16.7% of the participants.

Clinical examination revealed hallmark features of VLS in the majority of patients: depigmentation or skin whitening was observed in 92.3%, fissures in 83.3%, the classic “cigarette paper” atrophy in 66.7%, keratotic lesions in 25%, and either ecchymoses or blistering in 33%. Erosions were also present in 33% of cases. In 50% of the patients, lesions were confined to characteristic polar anatomical sites—namely, the 12:00 (supraclitoral) and 6:00 (perineal) positions. The remaining half of the cohort demonstrated more extensive involvement, with pathological changes affecting the entire vulvar region. The time from initial onset of symptoms to definitive diagnosis ranged from 1 to 18 months, with a mean diagnostic delay of 8.8 months.

In the next stage, selected peripheral blood hematological and biochemistry parameters were analyzed, and an immunophenotypic analysis was performed. The obtained test results were compared with those of healthy volunteers and are presented in Table 1.

**Table 1:** Tabular presentation of the results of hematological, biochemistry and immunophenotype of peripheral blood in patients with VLS and healthy volunteers (HV).

Parameters	VLS (n = 16)		HV (n = 16)		*p*-value
	Mean ± SD	Median (range)	Mean ± SD	Median (range)	
HGB [g/dl]	12.75 ± 1.38	12.7 (10.2–14.7)	11.47 ± 0.89	11.21 (10.13–13.23)	0.323
Hematocrit [%]	37.68 ± 3.77	37.55 (31.9–43.5)	36.02 ± 2.25	35.97 (32.09–38.80)	0.809
RBC [10^6^/µl]	4.68 ± 0.32	4.63 (4.18–5.11)	3.93 ± 0.68	3.80 (3.04–4.96)	0.491
MCV [fl]	80.5 ± 4.42	80.7 (71.2–86.2)	81.41 ± 4.14	81.48 (75.49–86.92)	0.616
MCH [pg]	27.25 ± 1.91	27.25 (22.8–30.1)	26.69 ± 1.11	26.69 (24.88–28.68)	0.838
MCHC [g/dL]	33.81 ± 0.84	33.80 (35.3–32)	32.69 ± 0.79	32.78 (31.06–33.97)	0.696
WBC [10^3^/µl]	6.85 ± 1.41	7.08 (5.01–8.97)	8.46 ± 2.39	9.42 (4.04–11.00)	0.287
NEU [10^3^/µl]	3.68 ± 1.02	3.59 (2.44–5.46)	4.70 ± 1.56	5.10 (1.02–6.67)	0.171
EOS [10^3^/µl]	0.42 ± 0.66	0.19 (0.05–2.4)	0.29 ± 0.1	0.3 (0.09–0.43)	0.073
BAS [10^3^/µl]	0.04 ± 0.01	0.04 (0.01–0.05)	0.3 ± 0.1	0.36 (0.11–0.46)	0.224
LYM [10^3^/µl]	2.54 ± 0.83	2.32 (1.71–4.4)	2.93 ± 0.73	2.87 (1.55–4.16)	0.897
MON [10^3^/µl]	0.52 ± 0.16	0.5 (0.27–0.86)	0.46 ± 0.14	0.47 (0.19–0.73)	0.590
PLT [10^3^/µl]	281.25 ± 66.23	285 (135–384)	283 ± 74.41	296.00 (151.00–374.00)	0.224
CRP [mg/l]	49.99 ± 6.09	50.31 (42.27–59.27)	2.70 ± 0.96	2.68 (1.26–4.79)	< 0.001*
CD45+ [%]	88.63 ± 4.33	88.30 (80.57–94.89)	96.35 ± 2.41	97.38 (91.45–98.98)	< 0.001*
CD3+ [%]	60.18 ± 6.18	58.42 (52.28–71.15)	73.12 ± 3.18	72.34 (67.20–78.16)	< 0.001*
CD19+ [%]	8.18 ± 2.04	7.60 (5.14–11.77)	12.24 ± 2.61	12.17 (6.83–16.60)	< 0.001*
CD4+ [%]	27.46 ± 5.38	27.91 (20.49–36.00)	42.88 ± 3.44	43.70 (36.21–47.33)	< 0.001*
CD8+ [%]	32.72 ± 6.98	32.61 (18.81–49.59)	30.25 ± 4.04	29.27 (23.06–38.30)	0.210

HGB—Hemoglobin; RBC—Red Blood Cells; MCV—Mean Corpuscular Volume; MCH—Mean Corpuscular Hemoglobin; MCHC—Mean Corpuscular Hemoglobin Concentration; WBC—White Blood Cells; NEU—Neutrophils; EOS—Eosinophils; BAS—Basophils; LYM—Lymphocytes; MON—Monocytes; PLT—Plateletsp; CRP—C-Reactive Protein; *statistically significant results.

In the analysis of peripheral blood hematological parameters, no statistically significant differences were noted between patients with VLS and the control group in terms of the number of erythrocytes (RBC), hemoglobin concentration (HGB), hematocrit (HCT), red blood cell indices (MCV, MCH, MCHC), total leukocyte count (WBC), neutrophils (NEU), eosinophils (EOS), basophils (BAS), lymphocytes (LYM), monocytes (MON), or platelet count (PLT). All these parameters were within the normal range for childhood and did not show significant deviations (*p* > 0.05).

The only significant biochemical difference between the groups was the concentration of C-reactive protein (CRP), which was significantly higher in patients with VLS compared to healthy volunteers (mean 49.99 ± 6.09 mg/L vs 2.70 ± 0.96 mg/L, *p* < 0.001). This result confirms the presence of an active inflammatory process in the course of VLS.

In terms of the immunophenotype of peripheral blood cells, significant differences were observed between the groups. In patients with VLS, there was an apparent decrease in the percentage of CD45⁺ cells (mean 88.63 ± 4.33%) compared to the control group (96.35 ± 2.41%; *p* =  < 0.001), which may indicate a relative decrease in the number of mature leukocytes. The percentages of T lymphocytes (CD3+ : 60.18 ± 6.18% vs. 73.12 ± 3.18%; *p* =  < 0.001), CD4+ helper lymphocytes (27.46 ± 5.38% vs. 42.88 ± 3.44%; *p* =  < 0.001) and CD19+ B lymphocytes (8.18 ± 2.04% vs 12.24 ± 2.61%; *p* =  < 0.001) were also significantly reduced compared to healthy volunteers. It is worth noting that the percentage of CD8+ cytotoxic lymphocytes did not differ significantly between groups (*p* = 0.210), which may indicate a relative compensation of the CD8+ population with a decrease in the number of CD4+ lymphocytes. As a measure of effect size for comparisons between two independent groups, Cohen’s d was applied. The complete results of this analysis are provided in Table S1.

The immunophenotypic profile indicates significant disturbances in the T and B lymphocyte subpopulations in patients with VLS, especially in the immunosuppression of the CD4+ population, which may reflect a chronic inflammatory process and ongoing mechanisms of immune response regulation. These results correspond with the observed increase in CRP, indicating a significant involvement of the immune system in the pathogenesis of the disease.

### The role of selected immunoregulatory checkpoints and pro-inflammatory cytokines in the immunopathogenesis of Vulvar Lichen Sclerosus

**Fig. 2:**
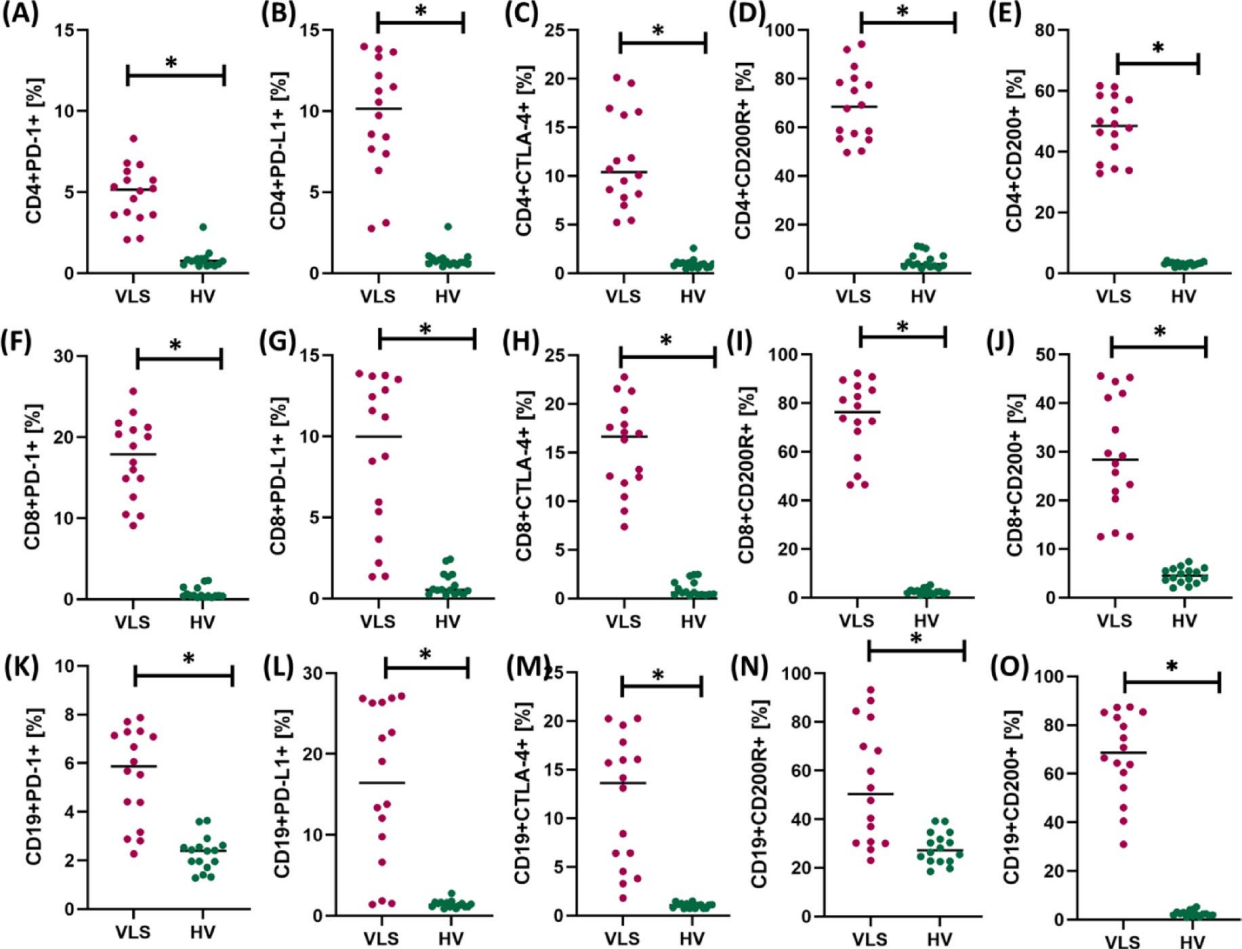
Graphical representation of changes in the percentage of occurrence of the tested immune checkpoints and their ligands on selected subpopulations of lymphocytes in patients with Vulvar Lichen Sclerosus (VLS) and healthy volunteers (HV). (**A**) shows the percentage of CD4⁺ T lymphocytes expressing PD-1, (**B**) the percentage of CD4⁺ T lymphocytes expressing PD-L1, (**C**) the percentage of CD4⁺ T lymphocytes expressing CTLA-4, (**D**) the percentage of CD4⁺ T lymphocytes expressing CD200R, and (**E**) the percentage of CD4⁺ T lymphocytes expressing CD200. Panels (**F**–**J**) present CD8⁺ T lymphocytes, where (**F**) shows the percentage of cells expressing PD-1, (**G**) PD-L1, (**H**) CTLA-4, (**I**) CD200R, and (**J**) CD200. The last group of panels (**K**–**O**) refers to CD19⁺ B lymphocytes, showing (**K**) the percentage of cells expressing PD-1, (**L**) PD-L1, (**M**) CTLA-4, (**N**) CD200R, and (**O**) CD200. Abbreviations: *statistically significant results; VLS—Vulvar Lichen Sclerosus; HV—healthy volunteers; PD-1—programmed cell death protein 1; PD-L1—programmed death-ligand 1; CTLA-4—cytotoxic T-lymphocyte-associated protein 4; CD200—cluster of differentiation 200; CD200R—CD200 receptor; CD4⁺—T helper lymphocytes; CD8⁺—cytotoxic T lymphocytes; CD19⁺—B lymphocytes.

Immune checkpoints with suppressor properties, such as CTLA-4, PD-1, or CD200R, and their ligands (e.g., PD-L1, CD200), play a key role in regulating the immune response. Therefore, in the next stage of our research, we decided to conduct a comparative analysis of the percentage of the mentioned molecules on selected subpopulations of T lymphocytes (CD4+ and CD8 +) and CD19+ B lymphocytes between newly diagnosed patients with VLS and healthy volunteers. The obtained test results are presented in Table 2 and Fig. 2.

**Table 2:** Tabular presentation of the results of the percentage of occurrence of the tested immune checkpoints and their ligands in patients with VLS and healthy volunteers (HV).

Parameters	VLS (n = 16)		HV (n = 16)		*p*-value
	Mean ± SD	Median (range)	Mean ± SD	Median (range)	
CD4+PD-1+ [%]	4.90 ± 1.67	5.15 (2.07–8.30)	0.85 ± 0.56	0.75 (0.45–2.84)	< 0.001*
CD4+PD-L1+ [%]	9.64 ± 3.47	10.15 (2.75–13.98)	0.85 ± 0.55	0.70 (0.41–2.87)	< 0.001*
CD4+CTLA-4+ [%]	11.58 ± 4.70	10.39 (5.22–20.11)	0.96 ± 0.48	0.91 (0.45–2.57)	< 0.001*
CD4+CD200R+ [%]	69.01 ± 14.16	68.45 (49.59–94.19)	5.20 ± 3.04	3.69 (1.95–11.09)	< 0.001*
CD4+CD200+ [%]	47.98 ± 9.76	48.43 (32.85–61.63)	3.09 ± 0.62	3.17 (1.90–4.14)	< 0.001*
CD8+PD-1+ [%]	17.31 ± 4.81	17.90 (9.09–25.67)	0.76 ± 0.67	0.45 (0.21–2.31)	< 0.001*
CD8+PD-L1+ [%]	8.75 ± 4.61	9.98 (1.34–13.87)	0.86 ± 0.70	0.53 (0.21–2.42)	< 0.001*
CD8+CTLA-4+ [%]	15.49 ± 4.50	16.65 (7.36–22.76)	1.00 ± 0.79	0.59 (0.26–2.48)	< 0.001*
CD8+CD200R+ [%]	73.42 ± 15.23	76.29 (46.38–92.28)	2.51 ± 1.12	2.36 (0.95–5.19)	< 0.001*
CD8+CD200+ [%]	29.30 ± 11.41	28.37 (12.52–45.58)	4.55 ± 1.52	4.50 (1.97–7.38)	< 0.001*
CD19+PD-1+ [%]	5.52 ± 1.88	5.87 (2.27–7.87)	2.29 ± 0.69	2.40 (1.28–3.64)	< 0.001*
CD19+PD-L1+ [%]	16.10 ± 9.50	16.44 (1.38–27.16)	1.41 ± 0.45	1.34 (0.85–2.77)	< 0.001*
CD19+CTLA-4+ [%]	11.72 ± 6.40	13.64 (1.81–20.26)	1.02 ± 0.25	1.07 (0.71–1.49)	< 0.001*
CD19+CD200R+ [%]	54.12 ± 23.47	50.34 (23.05–93.22)	28.17 ± 6.26	27.25 (18.47–39.20)	0.001*
CD19+CD200+ [%]	67.52 ± 17.05	68.62 (30.94–87.46)	2.51 ± 1.12	2.36 (0.95–5.19)	< 0.001*

*statistically significant results.

Immunophenotypic analysis revealed significantly increased expression of immune checkpoint molecules and their ligands on CD4+ T, CD8+ T, and CD19+ B lymphocyte subpopulations in patients with VLS compared to healthy volunteers. In the CD4⁺ population, a significantly higher percentage of cells expressing PD-1, PD-L1, CTLA-4, CD200, and CD200R was observed (*p* < 0.001 for all comparisons). Similarly, CD8⁺ lymphocytes from patients with VLS showed significantly higher expression of PD-1, PD-L1, CTLA-4, and molecules from the CD200/CD200R system (*p* < 0.001). Additionally, within the CD19+ B lymphocyte population, a statistically significant increase in the expression of all immune checkpoints tested (PD-1, PD-L1, CTLA-4, CD200, and CD200R; *p* ≤ 0.001) was observed. Cohen’s d for these analyses is presented in the Table S1. These results indicate a significant activation of immunosuppressive mechanisms in patients with VLS, which may be a compensatory response to chronic inflammation and reflect the phenomena of lymphocyte exhaustion and secondary immune tolerance.

**Fig. 3:**
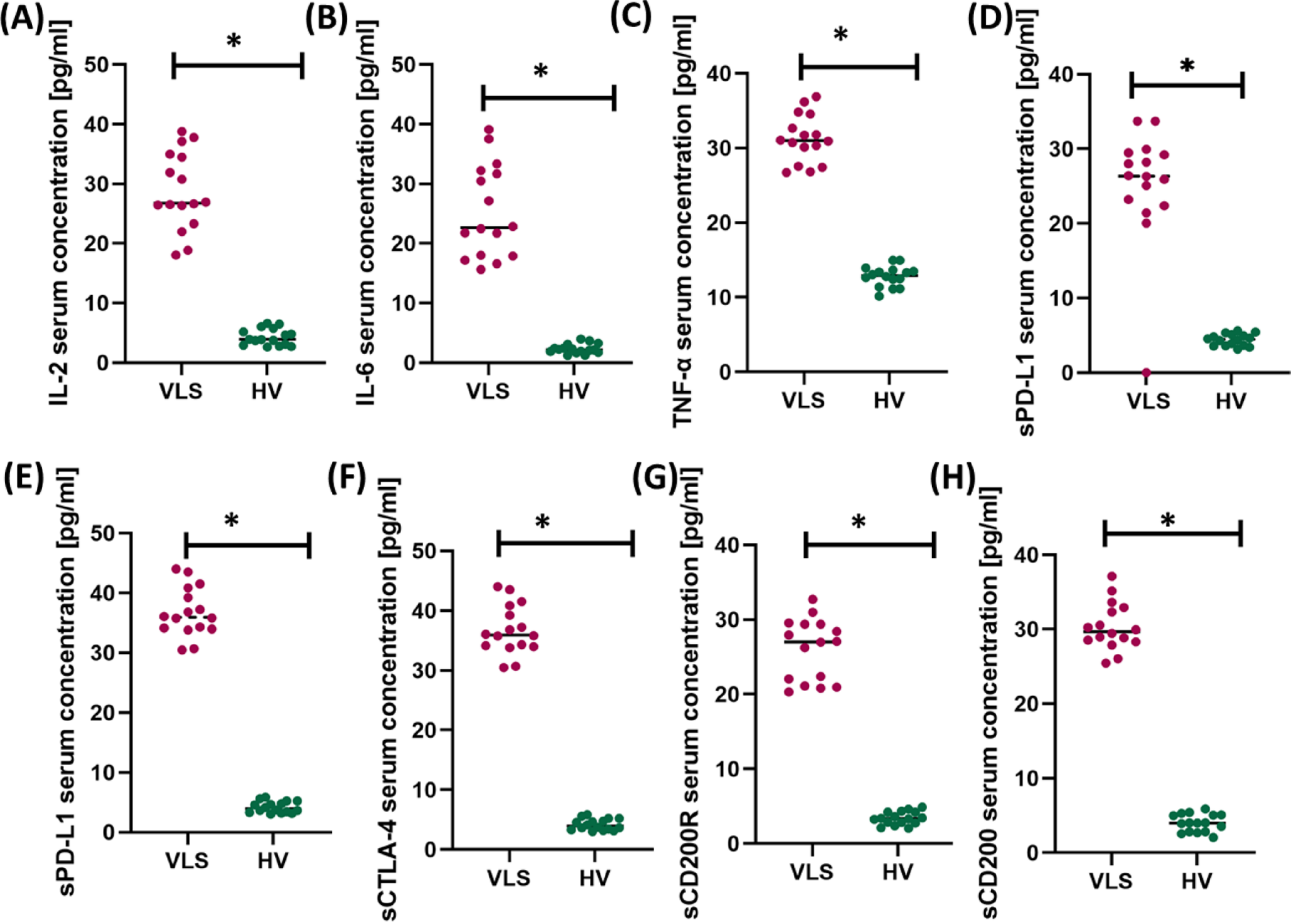
Graphical representation of changes in the serum concentration of the tested soluble forms of immune checkpoints and their ligands (**D**–**H**) and the concentration of proinflammatory cytokines (**A**–**C**) in patients with Vulvar Lichen Sclerosus (VLS) and healthy volunteers (HV). Panel (**A**) shows the serum concentration of interleukin-2 (IL-2), panel (**B**) shows the serum concentration of interleukin-6 (IL-6), and panel (**C**) shows the serum concentration of tumor necrosis factor alpha (TNF-α). Panel (**D**) presents the serum concentration of soluble programmed cell death protein 1 (sPD-1), panel (**E**) the serum concentration of soluble programmed death-ligand 1 (sPD-L1), panel (**F**) the serum concentration of soluble cytotoxic T-lymphocyte-associated protein 4 (sCTLA-4), panel (**G**) the serum concentration of soluble CD200 receptor (sCD200R), and panel (**H**) the serum concentration of soluble CD200 (sCD200). Data are presented as scatter plots with median values indicated, and asterisks (*) denote statistically significant differences between groups (*p* < 0.05). Abbreviations: VLS—Vulvar Lichen Sclerosus; HV—healthy volunteers; IL-2—interleukin-2; IL-6—interleukin-6; TNF-α—tumor necrosis factor alpha; sPD-1—soluble programmed cell death protein 1; sPD-L1—soluble programmed death-ligand 1; sCTLA-4—soluble cytotoxic T-lymphocyte-associated protein 4; sCD200R—soluble CD200 receptor; sCD200—soluble CD200.

Serum concentrations of interleukin-2 (IL-2), interleukin-6 (IL-6), and tumor necrosis factor alpha (TNF-α) were also measured, and the results are provided in Table 3 and Fig. 3.

**Table 3:** Tabular presentation of the results of serum concentration of the tested immune checkpoints and their ligands, and selected pro-inflammatory cytokines in patients with VLS and healthy volunteers (HV).

Parameters	VLS (n = 16)		HV (n = 16)		*p*-value
	Mean ± SD	Median (range)	Mean ± SD	Median (range)	
sPD-1 [pg/mL]	26.69 ± 3.88	26.36 (20.06–33.72)	4.40 ± 0.75	4.48 (3.16–5.60)	< 0.001*
sPD-L1 [pg/mL]	36.76 ± 3.97	35.95 (30.47–44.00)	4.20 ± 0.91	3.93 (3.01–5.84)	< 0.001*
sCTLA-4 [pg/mL]	43.96 ± 3.77	44.76 (36.08–48.24)	5.09 ± 1.09	4.84 (3.63–6.66)	< 0.001*
sCD200R [pg/mL]	26.02 ± 3.99	27.03 (20.31–32.70)	3.39 ± 0.84	3.34 (2.05–4.88)	< 0.001*
sCD200 [pg/mL]	30.33 ± 3.08	29.70 (25.44–37.11)	4.01 ± 1.15	4.00 (2.05–5.89)	< 0.001*
IL-2 [pg/mL]	28.80 ± 6.36	26.78 (18.07–38.77)	4.32 ± 1.33	3.92 (2.63–6.62)	< 0.001*
IL-6 [pg/mL]	25.35 ± 7.52	22.66 (15.64–39.12)	2.35 ± 0.78	2.20 (1.23–3.99)	< 0.001*
TNF-α [pg/mL]	31.26 ± 3.10	30.98 (26.71–36.88)	12.80 ± 1.30	12.91 (10.14–14.95)	< 0.001*

*statistically significant results.

In patients with VLS, significantly higher serum levels of all tested immune checkpoint molecules and their ligands were found compared to healthy volunteers (HV). Mean levels of soluble forms of PD-1, PD-L1, CTLA-4, CD200R, and CD200 were significantly increased (*p* <  < 0.001 for all comparisons), suggesting increased activation of immunoregulatory axes in the course of the disease. At the same time, a significant increase in the levels of proinflammatory cytokines IL-2, IL-6, and TNF-α was observed in the serum of patients with VLS (*p* < 0.001), indicating the presence of a systemic inflammatory component. Cohen’s d for these analyses is presented in the Table S1. These results confirm the involvement of both proinflammatory and suppressive immune mechanisms in the pathogenesis of VLS, suggesting the potential significance of the studied molecules as biomarkers of the active phase of the disease.

### Analysis of relationships using Spearman’s rank correlation

**Fig. 4:**
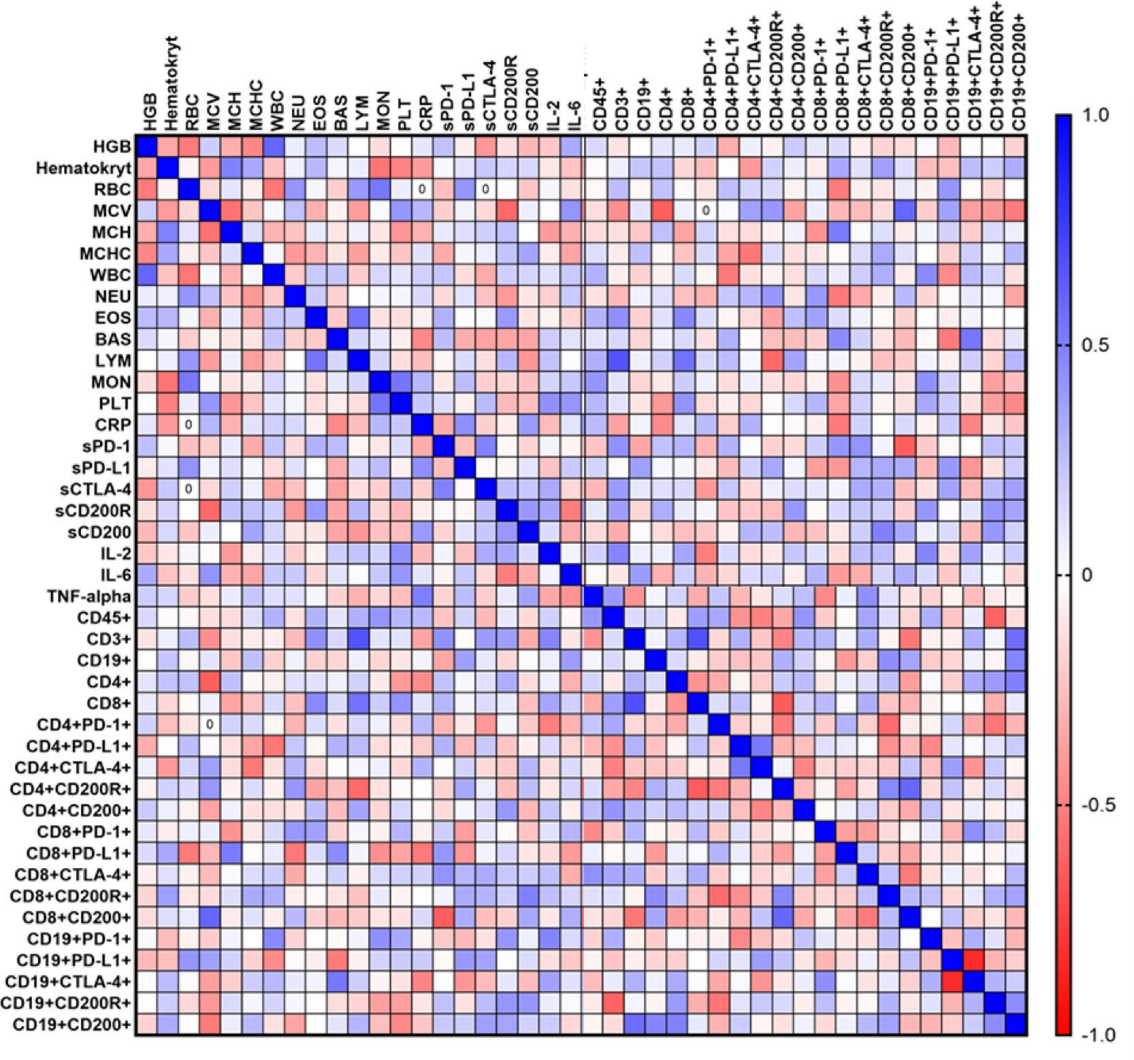
Graphical representation of Spearman’s rank correlation for VLS patients.

Due to several statistically significant changes observed in patients with VLS, in the next stage of the study, we decided to analyze the extent to which the percentage of occurrence of the tested immune checkpoints and their ligands as well as the concentrations of the tested cytokines, correlate with each other and with the parameters of the morphology and peripheral blood biochemistry of the tested patients. A graphical representation of the obtained results is shown in Fig. 4.

As a result of the analyses, we identified 41 statistically significant correlations, comprising 25 negative and 16 positive correlations (Table S2). The most critical negative correlations included CD19+ PD-L1+ & CD19+ CTLA-4, for which as PD-L1 expression on CD19+ B cells increases, CTLA-4 expression on the same cells decreases; CD8+ & CD4+ CD200R+ , for which a higher level of CD8+ lymphocytes is associated with a lower level of CD4+ lymphocytes expressing CD200R, and sPD-1 & CD8+ CD200+ , for which an increase in the level of sPD-1 is related to a decrease in the number of CD8+ lymphocytes expressing CD200. Moderate negative correlations between lymphocyte markers and blood parameters (e.g., MCV vs. CD4+) indicate potential interactions between inflammation and immune function. In the case of positive correlations, the following deserve special attention: CRP & TNF-α, for which an increase in the level of CRP protein is associated with an increase in the level of TNF-α, which indicates the co-occurrence of inflammation, and CD4+ PD-L1+ & CD4+ CTLA-4+ , for which an increase in the number of CD4+ lymphocytes expressing PD-L1 is associated with an increase in the number of CD4+ lymphocytes expressing CTLA-4, suggesting the co-occurrence of these markers in the immune response in the course of VLS.

## Discussion

### An overview of immunopathogenesis in VLS

Vulvar Lichen Sclerosus (VLS) is a chronic inflammatory dermatosis of unclear etiology; however, increasing evidence suggests a multifactorial pathogenesis, in which, in addition to genetic and hormonal factors, immune dysregulation plays a central role^1–7^. Numerous studies in adults have demonstrated that a hyperactive immune environment is characteristic of VLS lesions. The skin of the lesions contains dense T lymphocyte infiltrates (both CD4⁺ T helper cells and CD8⁺ cytotoxic cells) and a cytokine profile with a predominance of T helper 1 (Th1) cells—particularly high levels of interferon gamma (IFN-γ) and interleukin 2 (IL-2)—which are associated with keratinocyte damage, extracellular matrix degradation, and skin fibrosis^36,37^. Concomitantly, innate immune activation in VLS amplifies the inflammatory cycle: macrophages and other innate cells in the lesions produce proinflammatory cytokines such as tumor necrosis factor alpha (TNF-α) and IL-1β, which promote further leukocyte recruitment, increase the expression of adhesion molecules in the vascular endothelium, and drive connective tissue breakdown^38–40^. Antigen-presenting cells (especially dendritic cells) also contribute to the development of naive T cells in regional lymph nodes and direct them back to the skin, thus sustaining a self-perpetuating loop of chronic inflammation^33,34,39,41^. This immunopathological profile—a Th1-dependent T-cell attack on vulvar tissues accompanied by concomitant innate inflammatory factors—closely resembles other chronic inflammatory or autoimmune dermatoses, such as psoriasis and lichen planus, supporting the idea that immune dysregulation is crucial in VLS^1,9,17,22,26,33–35^.

### Paucity of immunological data in children

Although the immunopathogenesis of VLS in adults has been relatively well characterized, immunological studies of VLS in children are extremely sparse, making it difficult to extrapolate findings from adults to children. Farrell et al. (2006) provided some of the few pediatric-specific information by performing immunohistochemical studies of VLS lesions in prepubertal girls^42^. This study demonstrated increased expression of Th1/proinflammatory cytokines (IFN-γ, TNF-α, IL-1α) in the epidermis and upper dermis of children, along with increased expression of T cell activation markers such as CD25, intercellular adhesion molecule 1 (ICAM-1), CD11a, and HLA-DR^42^. This suggests that even in young patients, VLS elicits a strong cellular immune response, analogous to that in adults. However, beyond these isolated reports, pediatric data remain extremely limited. The paucity of immunological biomarker studies in children has been highlighted in the literature^42^, underscoring the need for specialized pediatric studies to elucidate disease mechanisms and enhance diagnosis and monitoring in this age group.

### Clinical differences between VLS in children and adults

From a clinical perspective, VLS in children is not simply “VLS in adults in a smaller body.” Children often present with different symptom patterns and risks, which have significant implications for diagnosis and treatment. For example, lichen sclerosus signs can be mistaken for vulvovaginitis, nonspecific vulvar irritation, or even sexual abuse in prepubertal patients. Orszulak et al. noted that prepubertal girls frequently report nonspecific symptoms—such as itching or burning, vulvar pain or dysuria, and constipation which are often mistaken for symptoms of other conditions typical for this age^10^, These factors contribute to frequent diagnostic delays in children^10^. It is also important to emphasize that although high-potency topical corticosteroids are the established first-line treatment at all ages, pediatric maintenance therapy and follow-up are not standardized, leading to a high relapse rate in girls with VLS^10^. An extensive, recent retrospective study by Liu et al. compared 97 girls (< 18 years) and 647 adult women with VLS, revealing several key epidemiological and clinical differences^43^:

Age of onset: In ~ 95% of affected girls, the disease began during prepuberty, whereas in most adult women, VLS developed during the mid-reproductive years (peak incidence ~ 75% during the reproductive years), and in only ~ 20% of adult women, the disease began after menopause^43^. In other words, VLS in children is primarily a prepubertal condition, whereas in adults, it most often manifests in middle age.

Pruritus (itching) was the most common symptom in both cohorts, but it was reported in almost all adult patients (~ 95%) compared to approximately 74% of pediatric patients^43^. In contrast, asymptomatic disease was more common in children (17.7% reported no symptoms) compared to only 2.2% of adults^43^. This suggests that a significant proportion of children may have unrecognized disease due to mild or absent symptoms, increasing the risk of delayed diagnosis. Classic porcelain-white patches and changes in vulvar architecture were typical in both cohorts. However, “mossy” or hypertrophic lesions and atrophy of the labia minora were more frequently observed in adult women. In contrast, in prepubertal patients, the perianal and labia majora were more frequently involved^43^. Adult women were more likely to have changes in the clitoris, vestibule, and labia minora (indicative of long-term scarring). In contrast, girls showed a greater tendency for whitening and fissuring in the perianal region. Squamous cell carcinoma (SCC) developing within lichen sclerosus is a recognized long-term risk of VLS in adults. In the series by Liu et al., eight adult patients (1.2%) developed secondary vulvar SCC, whereas no malignancy was reported among pediatric patients^43^. Pediatric VLS virtually never progresses to malignancy in childhood, a reassuring difference; however, it is unknown whether early-onset VLS may increase the risk of malignancy in later adulthood, emphasizing the need for vigilance. Although VLS primarily affects the genital skin, a small group of patients in the study (13 cases in total) had concomitant genital and extragenital LS lesions (most commonly on the trunk)^43^. Notably, the patients included 2 girls and 11 adults, indicating that extragenital symptoms, although rare, can occur even in children (though more frequently in adults). These clinical differences highlight the unique nature of VLS in children. Early diagnosis and appropriate treatment in children are crucial to prevent irreversible scarring and anatomical changes. Long-term surveillance is also essential—even if the immediate risk of malignancy is minimal in childhood, continued observation can ensure prompt treatment of any persistent or recurrent disease, mitigating future complications^43^. In short, clinicians should maintain a high level of suspicion for VLS in girls with chronic vulvar irritation or anatomical changes. Once diagnosed, aggressive treatment and long-term monitoring are recommended, rather than assuming that treatment can be based on adult protocols.

### New immunological discoveries in pediatric VLS

Our recent pediatric case series was one of the first studies to simultaneously evaluate inflammatory cytokines and immunoregulatory checkpoint molecules in children with VLS. We found that prepubertal VLS is characterized by a combination of markers of immune activation and immune regulation, suggesting a persistent imbalance rather than uncontrolled overactivation of the immune system. Blood samples from our pediatric cohort showed elevated levels of proinflammatory cytokines (including IL-2, IL-6, and TNF-α) along with increased expression of inhibitory immune checkpoints such as PD-1/PD-L1, CTLA-4, and the CD200/CD200R pathway. This indicates that the pediatric immune system is strongly activated against vulvar tissues (reflecting the cytokine environment), but simultaneously attempts to control this inflammatory state by increasing the expression of suppressive signals. This pattern is consistent with the concept of a dysregulated immune response—the body intensifies its inflammatory attack on the skin, while counterregulatory mechanisms are simultaneously, albeit insufficiently, activated to control this attack. Importantly, our finding of increased expression of checkpoint molecules in VLS tissue is a novel discovery, particularly in pediatric patients. It raises interesting parallels with observations in other contexts. For example, immune checkpoint inhibitor (ICI) therapies used in oncology—drugs that block PD-1, PD-L1, or CTLA-4 to release antitumor T cells—can induce lichenoid dermatoses (lesions resembling lichen planus or lichen sclerosus) as a side effect of increased immune system activity. A recent systematic review of cancer patients treated with ICI inhibitors found that approximately 21% of immune-related cutaneous adverse events were lichenoid eruptions identified as LS, with the majority of cases responding to immunosuppressive therapy^44^

In other words, when the PD-1/PD-L1 or CTLA-4 pathways are pharmacologically blocked, a proportion of patients develop lichen sclerosus, likely because previously controlled autoreactive T cells become overactive. This observation from oncology indirectly supports the notion that the PD-1 and CTLA-4 pathways usually function to limit the type of T-cell-mediated damage observed in VLS. Therefore, increased expression of PD-1, PD-L1, and CTLA-4 in pediatric VLS lesions may reflect the immune system’s attempt to balance and limit chronic inflammation. It’s a dynamic tug-of-war: pro-inflammatory forces drive the disease, while checkpoint signals simultaneously attempt to suppress this inflammation. As a result, VLS, an insufficiently controlled immune attack causes ongoing tissue damage. Notably, the Th1-biased inflammatory profile we identified in children (characterized by elevated levels of IL-2, TNF-α, etc.) reflects the immunological environment documented in adult disease^1,9^. This indicates that, at a fundamental level, VLS in children and adults shares common immunopathogenic threads, primarily chronic inflammation driven by T lymphocytes. Our pediatric patients showed many of the same elevated cytokine levels observed in adult studies (e.g., high levels of TNF-α and IL-6 in lesions, as reported by Tran et al.^1^). This association suggests that children, like adults, generate a strong Th1-type immune response in VLS. On the other hand, the concomitant high expression of inhibitory checkpoints (PD-1/PD-L1, CTLA-4, CD200/CD200R) in our pediatric cohort represents an additional aspect that has been less explored in adults with VLS. This suggests that the more adaptive immune system of children may more strongly engage regulatory pathways, perhaps to a greater extent than what is typically observed in adult-onset disease, to balance inflammation. Whether adults with VLS also show similar checkpoint expression remains open, as this has not been systematically investigated in older populations.

### Immune mechanisms and clinical correlates

Understanding the immune environment of VLS helps explain many of the disease’s clinical manifestations. Elevated levels of IL-1β, TNF-α, and IL-6 in the skin may have a neurosensitizing effect. These proinflammatory cytokines are known to stimulate peripheral nociceptive fibers, which may contribute to the intense pruritus (itching) and burning sensations often experienced by patients^45^. Indeed, inflammatory cytokines not only damage tissue but also directly trigger or amplify itch and pain signaling pathways in the skin. Furthermore, chronic inflammation in VLS leads to barrier dysfunction and tissue remodeling. Cytokine-induced keratinocyte stress, combined with the breakdown of the extracellular matrix by proteases (resulting from increased expression in the inflammatory environment), leads to the fragility of the vulvar skin. This corresponds to the clinical observation of fissures, erosions, and easily ruptured skin in active disease^38–40^. Over time, the ongoing cycle of inflammation and subsequent fibrosis leads to the development of the classic porcelain-white atrophic plaques and scarring (loss of standard vulvar architecture) characteristic of long-term VLS. In short, immunopathological factors (Th1 cytokines, TNF-α, etc.) drive symptoms such as itching/pain and directly cause physical symptoms such as whitened, thickened, or thinned patches and scars.

Although our pediatric study was not large enough to obtain statistically significant correlations between individual biomarker levels and clinical severity, the trends we observed are consistent with disease activity: patients with more visible lesions tended to have higher expression of immune markers. This suggests that immune biomarkers may potentially reflect disease burden. For example, a child with severely elevated tissue levels of TNF-α or IL-6 may have more extensive or symptomatic disease. This is speculative, but it provides a basis for further investigation. Ultimately, the immune profile appears consistent with the clinical presentation of VLS—active inflammation correlates with symptoms and visible skin lesions, supporting the idea that measuring these immune mediators can help assess disease activity. This also illustrates how VLS essentially creates a self-perpetuating inflammatory loop: inflammation causes tissue damage and symptoms, which in turn can further stimulate the immune response (for example, scratching due to itching can worsen skin damage and antigen release, driving greater immune activation). Without intervention, this loop can become a chronic condition. Given the evidence of immune system involvement, this supports the theory that VLS behaves similarly to an autoimmune condition, in which the body’s immune response is unable to shut down effectively.

### Therapeutic implications of immune dysregulation

The recognition of immune dysregulation in VLS opens the door to targeted therapeutic strategies beyond conventional corticosteroid therapy. Currently, ultrapotent topical corticosteroids (such as clobetasol propionate) are the first-line treatment for VLS of all ages. They are effective in controlling symptoms and slowing disease progression in most patients. However, steroids represent a nonspecific anti-inflammatory approach. The cytokine profile observed in VLS has sparked interest in more specific interventions. For example, persistently elevated levels of TNF-α and IL-6 have been observed in cutaneous lesions (and even systemically) in VLS^1^. These cytokines are key drivers of chronic inflammation and fibrosis. They are targets of existing biologics used in other diseases (such as anti-TNF therapies in psoriasis/arthritis and anti-IL-6 therapies in rheumatoid arthritis). Some authors speculate that biologics targeting TNF-α or IL-6 may provide therapeutic benefit in refractory cases of lichen sclerosus^1^. Although this approach remains experimental, and no targeted therapy has yet become standard for the treatment of VLS, it represents a logical direction for future research or clinical trials—particularly in patients who do not respond adequately to steroids or who require long-term steroid-sparing therapy. Beyond cytokines, there is growing evidence of an autoimmune component in VLS. The presence of autoantibodies against extracellular matrix protein 1 (ECM1) in many VLS patients (reported in approximately 74% of women with VLS in one study) also suggests a B-cell-mediated aspect of the disease^9^. ECM1 is a structural protein of the dermis, and antibodies against it may contribute to tissue damage. This finding further supports the classification of VLS as an autoimmune disorder and suggests the use of systemic immunomodulatory drugs in severe cases. Corazza et al. reviewed the immunopathogenesis of VLS and concluded that immune-targeted therapies warrant investigation^9^. They highlighted, for example, the role of IL-2 (a T-cell growth factor elevated in VLS lesions) in promoting Th1 responses and emphasized the need for clinical trials of immune-targeted therapies (whether biologics, immunomodulators, or other novel agents) to improve VLS treatment outcomes^9^. Particularly in pediatric patients, our findings regarding checkpoint regulation suggest that the immune system attempts to self-regulate. An intriguing, though difficult to understand, implication is whether we could therapeutically enhance regulatory pathways or otherwise tilt the balance in favor of immunoregulation. Although directly enhancing PD-1/PD-L1 or CTLA-4 signaling is not clinically feasible with currently available drugs (indeed, as noted, blocking these checkpoints can induce LS-like inflammation), this knowledge still has practical value. This suggests that aggressive immunosuppression may not be the only approach; promoting immune tolerance may be another goal. Practically speaking, this means that if a child’s VLS is not adequately controlled with topical steroids, physicians should consider early introduction of second-line therapies (such as topical calcineurin inhibitors like tacrolimus, or short courses of systemic immunosuppressants in extreme cases) to suppress inflammation before significant scarring occurs. Our data suggest that we should maintain a lower threshold for adjunctive therapy or referral to a specialist for pediatric VLS when there are signs (clinical or via potential biomarkers) of an ongoing active immune response despite standard treatment. Early intervention with additional immunomodulatory strategies can not only alleviate current symptoms but also potentially reduce long-term tissue damage. Furthermore, given that children have many years of life ahead of them, treating VLS as a chronic disease becomes crucial. This requires proactive, long-term management: even after achieving initial disease control, children should be placed on a maintenance treatment plan (e.g., low-dose topical corticosteroids, calcineurin inhibitors, or other treatment regimens as needed) and regularly monitored. Caregiver education is also crucial to ensure that subtle signs of relapse are quickly recognized and treated. Indeed, VLS in children may benefit from a more aggressive and long-term approach than that which could be used alone.

### Study limitations and future research

Despite the promising nature of our results, which identify potential immune biomarkers in pediatric VLS, it is essential to acknowledge the limitations of our study (and current pediatric data in general) before overinterpreting these findings. Due to the small sample size and the rarity of the VLS, our study included only a limited number of patients. This small cohort size may not accurately reflect the full heterogeneity of the disease, limiting the statistical power to draw definitive conclusions. Second is demographic homogeneity. All patients in our report were from a relatively homogeneous ethnic and geographic background. The immune profile of VLS may differ in other populations. Therefore, our results may not be generalizable to all pediatric patients with VLS (for example, there may be genetic or environmental differences influencing the immune response). The next fact is that the immune markers were measured at a single time point for each patient. VLS is a dynamic disease, and immune marker levels may fluctuate throughout the course of the disease or during treatment. Due to the cross-sectional clinical presentation, we were unable to assess how these biomarkers change over the course of disease progression or improvement (e.g., before treatment versus after treatment). Also, our data suggest associations (e.g., higher cytokine levels in VLS lesions), but do not prove that these immunological changes are the cause of VLS. The immunological changes may be a consequence of another primary factor. We also cannot rule out the possibility that some unmeasured factor drives both the immune response and disease symptoms. The last one is biomarker specificity; the molecules we identified as elevated (such as PD-1, CTLA-4, CD200/CD200R, IL-2, IL-6, and TNF-α) are part of broad immunological pathways. Many of these are not specific to VLS—for example, other chronic inflammatory skin diseases may also exhibit elevated levels of TNF or IL-6. This limits their usefulness as standalone diagnostic biomarkers for VLS. A single cytokine or checkpoint molecule is unlikely to distinguish VLS from other conditions without additional context. Given these limitations, our results should be considered hypotheses rather than conclusions. They highlight specific immunological pathways that warrant further investigation in larger cohorts. There is a clear need for multicenter studies and long-term follow-up of VLS in children in the future. Collaborative efforts could include a larger and more diverse sample of patients, which would help confirm whether the immunological patterns we observed are broadly representative. The inclusion of control groups (both healthy individuals and individuals with other inflammatory skin diseases) will be crucial in determining which biomarkers, or combinations thereof, are truly characteristic of VLS. Future studies would also benefit from a longitudinal design for example, tracking immune marker levels in the same child during treatment and remission. Such data could reveal whether certain cytokine levels decrease when the disease is controlled (making them useful for monitoring) or whether the expression of certain checkpoints increases after therapy (indicating restored immune regulation). We also propose exploring combinatorial biomarker panels. Rather than relying on a single marker, a specific signature composed of multiple immune molecules could increase the diagnostic specificity of VLS. For example, the simultaneous presence of elevated Th1 cytokines (such as IL-2) along with high expression of inhibitory receptors (such as PD-1 on affected T cells) could create a pattern that differentiates VLS from other dermatoses. Assessing such profiles across diseases could identify a unique immune fingerprint for lichen sclerosus. Furthermore, future studies should include pediatric patients at multiple time points, including baseline (off treatment), during active treatment, and in remission, to determine how interventions affect the immune system. This would also help answer the question of whether immunological changes are a cause or consequence of the disease (for example, if levels of a given cytokine normalize after successful treatment, this strengthens the argument that it was directly involved in disease activity).

## Methods

### Characteristics of patients and biological material

Based on the inclusion and exclusion criteria, patients aged 2 to 18 years with a clinically confirmed diagnosis of VLS were qualified for the study. The condition for participation was to obtain informed consent from a legal representative, and in the case of patients over 13 years of age, also the permission of the participant herself. The study excluded individuals who had received local glucocorticosteroids or immunomodulatory drugs in the vulva area within the last 3 months, as well as those who had taken systemic immunosuppressive or anti-inflammatory drugs during the same period. Additional exclusion criteria included: presence of chronic or acute infections and other pathologies of the vulva or vagina, acute systemic infection (viral, bacterial, or fungal), congenital or acquired immunodeficiencies, severe allergic reactions, autoimmune diseases, history of bone marrow transplantation, health condition preventing informed consent to participate in the study, and pregnancy or lactation. The control group consisted of healthy girls of similar age, without clinical features of inflammatory, immunological, or dermatological diseases, meeting analogous inclusion and exclusion criteria, with the exclusion of VLS diagnosis. The study cohort comprised 16 treatment-naïve female patients with newly diagnosed VLS, aged between 3.75 and 17.7 years (mean age: 6.78 ± 2.15 years; median: 9 years), along with 16 age-matched healthy female controls (mean age: 7.83 ± 4.4 years; median: 6 years; age range: 4–9 years). The recorded BMI values were within the normal range for age in both groups, and no cases of overweight or obesity were observed. Therefore, although BMI and pubertal stage were not variables actively matched during recruitment, they were documented and did not differ in a way that could influence the interpretation of immunological parameters in our study. The biological material for analysis was collected from the basilic vein as peripheral blood. A total of 10 mL of blood was collected from each participant: 5 mL was placed in tubes for clotting, and 5 mL was placed in tubes with an appropriate reagent, enabling the preparation of serum. The collected samples were used for further immunological analyses, including determinations of cytokines and immune checkpoints.

### Immunophenotyping with assessment of the expression of immune checkpoints

The assessment of the immunophenotype of lymphocytes and the evaluation of the expression of the examined checkpoints were performed on peripheral blood samples using flow cytometry. Blood samples were incubated with fluorochrome-conjugated anti-human monoclonal antibodies (anti-CD45 FITC, anti-CD3 BV510, anti-CD4 BV650, anti-CD8 BV605, anti-CD19 PerCp, anti-PD-1 PE, anti-PD-L1 APC, anti-CTLA-4 PE, anti-CD200 APC, and anti-CD200R PE BioLegend (San Diego, CA, USA)). After incubation with antibodies, the sample was lysed to remove erythrocytes using commercial BD lysis buffer (Franklin Lakes, New Jersey, USA). Then, the lysed sample was washed three times in Staining Buffer BD (Franklin Lakes, New Jersey, USA). The samples thus prepared were analyzed on a flow cytometer, CytoFLEX LX (Beckman Coulter, Indianapolis, IN, USA). The gating strategy for immune checkpoints was based on FMO. The data thus obtained were analyzed using Kaluza Analysis software (Beckman Coulter, Indianapolis, USA). At the same time, to maintain quality and consistency, the technical condition of the cytometer was assessed before sample analysis using CytoFLEX Ready-to-Use Daily QC Fluophores reagents (Beckman Coulter, Indianapolis, USA).

### Assessment of the concentration of soluble forms of immune checkpoints and their ligands, and the concentration of pro-inflammatory cytokines in the serum of patients with VLS

ELISA analyses were performed for soluble forms of sPD-1, sPD-L1, sCTLA-4, sCD200R, sCD200, IL-2, IL-6, and TNF-α using commercial ELISA kits according to the manufacturer’s instructions. The serum was diluted according to the kit instructions. Standards were prepared by a series of dilutions, creating a calibration curve. Absorbance was measured at 450 nm using a Victor 3 microplate reader (PerkinElmer). A calibration curve was prepared based on the standards, and the concentrations of analytes in the samples were determined based on this curve. The sensitivity ranges of the ELISA kits were as follows: PD-1 Human ELISA Kit (Thermo Fisher Scientific, cat. no. BMS2214): 2.34–150 pg/mL (analytical sensitivity 1.14 pg/mL); PD-L1 Human ELISA Kit (Thermo Fisher Scientific, cat. no. BMS2327): 4.69–300 pg/mL (analytical sensitivity < 0.6 pg/mL); CTLA-4 Human ELISA Kit (Thermo Fisher Scientific, cat. no. BMS276): 0.16–10.0 ng/mL (analytical sensitivity 0.13 ng/mL); CD200R Human ELISA Kit (Thermo Fisher Scientific, cat. no. EH510RB): 0.01–10 ng/mL (analytical sensitivity 0.01 ng/mL); CD200 Human ELISA Kit (Thermo Fisher Scientific, cat. no. EHCD200): 24.58–6000 pg/mL (analytical sensitivity 20 pg/mL); Human IL-2 ELISA Kit (Thermo Fisher Scientific, cat. no. BMS221-2): 18.8–1200 pg/mL (analytical sensitivity 9.1 pg/mL); Human IL-6 ELISA Kit (Thermo Fisher Scientific, cat. no.EH2IL6): 10.24–400 pg/mL (analytical sensitivity < 1 pg/mL); and Human TNF-α ELISA Kit (Thermo Fisher Scientific, cat. no. KHC3011): 15.6–1000 pg/mL (analytical sensitivity 1.7 pg/mL).

### Statistical analysis of the obtained research results

Statistical analyses included the Mann–Whitney U test, Spearman rank correlations, and ROC curve analyses. The Mann–Whitney U test was used to compare differences between two independent groups for nonparametric variables. This test assesses whether the distributions of the two groups differ from each other, assuming that the samples are independent and that the data are nonparametric. Results are presented as medians with interquartile ranges, and statistical significance was determined at *p* < 0.05. Spearman’s rank correlations were used to assess the strength and direction of the relationship between two variables. The correlation results were presented as correlation coefficients with the corresponding *p*-values. For post hoc power analysis, we used Cohen’s d, a standard measure of effect size for comparisons of two independent groups. This metric enables standardization of results obtained across different measurement scales (e.g., cytokines, cellular markers) and is widely recommended as the basis for calculating statistical power. All statistical analyses were performed using Statistica version 13.5.0.17 statistical software (TIBCO Software Inc.). The results were presented in tables and graphs for data visualization using GraphPad software (GraphPad Software, LLC).

## Conclusion

VLS in prepubescent girls poses a complex clinical and immunological challenge. Research indicates that chronic inflammation and excessive immune system activation are key elements in the pathogenesis of this disease. Excess interleukins and the increased expression of immune checkpoints such as PD-1, CD200R, and CTLA-4, as indicated by our study, may contribute to chronic inflammation and tissue damage observed in VLS. Further research should focus on thoroughly understanding the implications of these preliminary results in the molecular and cellular mechanisms that underlie VLS. In particular, it is vital to investigate the role of various interleukins and cytokines in the induction and maintenance of chronic inflammation. In parallel, the influence of checkpoints such as PD-1, CD200R, and CTLA-4 on regulating the immune response in the context of VLS requires investigation. Identifying key signaling pathways may open new directions for targeted therapies. Moreover, developing specific biomarkers may significantly improve the diagnosis and monitoring of VLS progression. These biomarkers could include specific interleukins, cytokines, or checkpoint expression levels. Additionally, these biomarkers could help predict response to various therapeutic interventions, allowing for a more personalized approach to treatment. Current therapeutic options for VLS are limited and often ineffective in the long-term management of the disease. Future therapies could focus on modulating the immune response to reduce chronic inflammation without inducing excessive immunosuppression.

## Supplementary Information

Below is the link to the electronic supplementary material.

Supplementary Material 1

## Data Availability

The data sets generated and/or analyzed during the current study are included within the manuscript or supplementary information files. Additional data are available from the first author upon reasonable request.
