# Supplementary material for: Elevated expression of immune checkpoints and pro-inflammatory cytokines as potential biomarkers in pediatric Vulvar Lichen Sclerosus
Source: Sci Rep. 2026 Feb 2;16:4543. doi: 10.1038/s41598-025-33630-2 (PMC12867991; doi:10.1038/s41598-025-33630-2)
Supplement: Supplementary file 1 — Supplementary Material 1 [file 41598_2025_33630_MOESM1_ESM.docx]

**Table S1:** Post hoc effect size analysis (Cohen’s d).

| **Parameter** | **Mean (VLS)** | **SD (VLS)** | **Mean (HV)** | **SD (HV)** | **Cohen’s d** | **Effect size** | **Reported p** |
| --- | --- | --- | --- | --- | --- | --- | --- |
| HGB [g/dl] | 12.75 | 1.38 | 11.47 | 0.89 | 1.09 | large | 0.323 |
| Hematocrit [%] | 37.68 | 3.77 | 36.02 | 2.25 | 0.53 | medium | 0.809 |
| RBC [10^6/µl] | 4.68 | 0.32 | 3.93 | 0.68 | 1.30 | very large | 0.491 |
| MCV [fl] | 80.50 | 4.42 | 81.41 | 4.14 | -0.21 | small | 0.616 |
| MCH [pg] | 27.25 | 1.91 | 26.69 | 1.11 | 0.34 | small | 0.838 |
| MCHC [g/dL] | 33.81 | 0.84 | 32.69 | 0.79 | 1.38 | very large | 0.696 |
| WBC [10^3/µl] | 6.85 | 1.41 | 8.46 | 2.39 | -0.82 | large | 0.287 |
| NEU [10^3/µl] | 3.68 | 1.02 | 4.70 | 1.56 | -0.77 | medium | 0.171 |
| EOS [10^3/µl] | 0.42 | 0.66 | 0.29 | 0.10 | 0.26 | small | 0.073 |
| BAS [10^3/µl] | 0.04 | 0.01 | 0.30 | 0.10 | -3.45 | very large | 0.224 |
| LYM [10^3/µl] | 2.54 | 0.83 | 2.93 | 0.73 | -0.49 | small | 0.897 |
| MON [10^3/µl] | 0.52 | 0.16 | 0.46 | 0.14 | 0.40 | small | 0.590 |
| PLT [10^3/µl] | 281.25 | 66.23 | 283.00 | 74.41 | -0.02 | trivial | 0.224 |
| **CRP [mg/L]** | 49.99 | 6.09 | 2.70 | 0.96 | 9.04 | very large | <0.001* |
| **CD45+ [%]** | 88.63 | 4.33 | 96.35 | 2.41 | -2.12 | very large | <0.001* |
| **CD3+ [%]** | 60.18 | 6.18 | 73.12 | 3.18 | -2.55 | very large | <0.001* |
| **CD19+ [%]** | 8.18 | 2.04 | 12.24 | 2.61 | -1.70 | very large | <0.001* |
| **CD4+ [%]** | 27.46 | 5.38 | 42.88 | 3.44 | -3.23 | very large | <0.001* |
| CD8+ [%] | 32.72 | 6.98 | 30.25 | 4.04 | 0.42 | small | 0.210 |
| CD4⁺PD-1⁺ [%] | 4.90 | 1.67 | 0.85 | 0.56 | 3.20 | very large | <0.001* |
| CD4⁺PD-L1⁺ [%] | 9.64 | 3.47 | 0.85 | 0.55 | 3.11 | very large | <0.001* |
| CD4⁺CTLA-4⁺ [%] | 11.58 | 4.70 | 0.96 | 0.48 | 2.77 | very large | <0.001* |
| CD4⁺CD200R⁺ [%] | 69.01 | 14.16 | 5.20 | 3.04 | 6.09 | very large | <0.001* |
| CD4⁺CD200⁺ [%] | 47.98 | 9.76 | 3.09 | 0.62 | 6.05 | very large | <0.001* |
| CD8⁺PD-1⁺ [%] | 17.31 | 4.81 | 0.76 | 0.67 | 4.11 | very large | <0.001* |
| CD8⁺PD-L1⁺ [%] | 8.75 | 4.61 | 0.86 | 0.70 | 2.19 | very large | <0.001* |
| CD8⁺CTLA-4⁺ [%] | 15.49 | 4.50 | 1.00 | 0.79 | 3.92 | very large | <0.001* |
| CD8⁺CD200R⁺ [%] | 73.42 | 15.23 | 2.51 | 1.12 | 6.09 | very large | <0.001* |
| CD8⁺CD200⁺ [%] | 29.30 | 11.41 | 4.55 | 1.52 | 2.73 | very large | <0.001* |
| CD19⁺PD-1⁺ [%] | 5.52 | 1.88 | 2.29 | 0.69 | 2.06 | very large | <0.001* |
| CD19⁺PD-L1⁺ [%] | 16.10 | 9.50 | 1.41 | 0.45 | 2.08 | very large | <0.001* |
| CD19⁺CTLA-4⁺ [%] | 11.72 | 6.40 | 1.02 | 0.25 | 2.19 | very large | <0.001* |
| CD19⁺CD200R⁺ [%] | 54.12 | 23.47 | 28.17 | 6.26 | 1.54 | very large | 0.001* |
| CD19⁺CD200⁺ [%] | 67.52 | 17.05 | 2.51 | 1.12 | 5.02 | very large | <0.001* |
| sPD-1 [pg/ml] | 26.69 | 3.88 | 4.40 | 0.75 | 7.08 | very large | <0.001* |
| sPD-L1 [pg/ml] | 36.76 | 3.97 | 4.20 | 0.91 | 8.62 | very large | <0.001* |
| sCTLA-4 [pg/ml] | 43.96 | 3.77 | 5.09 | 1.09 | 10.66 | very large | <0.001* |
| sCD200R [pg/ml] | 26.02 | 3.99 | 3.39 | 0.84 | 7.16 | very large | <0.001* |
| sCD200 [pg/ml] | 30.33 | 3.08 | 4.01 | 1.15 | 10.16 | very large | <0.001* |
| IL-2 [pg/ml] | 28.80 | 6.36 | 4.32 | 1.33 | 5.02 | very large | <0.001* |
| IL-6 [pg/ml] | 25.35 | 7.52 | 2.35 | 0.78 | 4.11 | very large | <0.001* |
| TNF-α [pg/ml] | 31.26 | 3.10 | 12.80 | 1.30 | 6.96 | very large | <0.001* |

**Table S2:** Detailed analysis of the obtained Spearman rank correlations for patients with VLS

| A pair of variables | R | t(N-2) | p |
| --- | --- | --- | --- |
| CD19+PD-L1+ & CD19+CTLA-4+ | -0.829 | -5.555 | 0.000 |
| CD8+ & CD4+CD200R+ | -0.635 | -3.078 | 0.008 |
| sPD-1 & CD8+CD200+ | -0.632 | -3.054 | 0.009 |
| MCV & CD4+ | -0.629 | -3.031 | 0.009 |
| CD45+ & CD19+CD200R+ | -0.609 | -2.872 | 0.012 |
| MCV & sCD200R | -0.591 | -2.743 | 0.016 |
| LYM & CD4+CD200R+ | -0.582 | -2.680 | 0.018 |
| CD4+PD-1+ & CD8+CD200R+ | -0.575 | -2.633 | 0.020 |
| MCV & MCH | -0.550 | -2.464 | 0.027 |
| Hematokryt & MON | -0.541 | -2.408 | 0.030 |
| CD4+PD-1+ & CD19+CD200R+ | -0.537 | -2.383 | 0.032 |
| RBC & WBC | -0.535 | -2.371 | 0.033 |
| NEU & CD8+PD-L1+ | -0.531 | -2.346 | 0.034 |
| CD3+ & CD8+CD200+ | -0.529 | -2.335 | 0.035 |
| MCHC & CD4+CTLA-4+ | -0.526 | -2.317 | 0.036 |
| WBC & CD4+PD-L1+ | -0.526 | -2.317 | 0.036 |
| CRP & CD8+PD-L1+ | -0.524 | -2.299 | 0.037 |
| BAS & CD19+PD-L1+ | -0.521 | -2.281 | 0.039 |
| HGB & RBC | -0.518 | -2.264 | 0.040 |
| RBC & CD8+PD-L1+ | -0.518 | -2.264 | 0.040 |
| MCV & CD19+CD200+ | -0.518 | -2.264 | 0.040 |
| CD8+CTLA-4+ & CD8+CD200+ | -0.518 | -2.264 | 0.040 |
| CD4+PD-1+ & CD4+CD200R+ | -0.514 | -2.240 | 0.042 |
| IL-2 & CD4+PD-1+ | -0.509 | -2.214 | 0.044 |
| sCD200R & IL-6 | -0.509 | -2.212 | 0.044 |
| CRP & TNF-alpha | 0.500 | 2.160 | 0.049 |
| MCH & CD8+PD-L1+ | 0.512 | 2.229 | 0.043 |
| CD4+PD-L1+ & CD4+CTLA-4+ | 0.518 | 2.264 | 0.040 |
| RBC & MON | 0.524 | 2.299 | 0.037 |
| BAS & CD19+CTLA-4+ | 0.532 | 2.353 | 0.034 |
| EOS & LYM | 0.541 | 2.408 | 0.030 |
| LYM & EOS | 0.541 | 2.408 | 0.030 |
| MON & PLT | 0.541 | 2.408 | 0.030 |
| PLT & MON | 0.541 | 2.408 | 0.030 |
| LYM & CD8+ | 0.550 | 2.464 | 0.027 |
| CD3+ & CD19+CD200+ | 0.568 | 2.580 | 0.022 |
| CD4+CD200R+ & CD8+CD200+ | 0.591 | 2.743 | 0.016 |
| HGB & WBC | 0.600 | 2.806 | 0.014 |
| MCV & CD8+CD200+ | 0.600 | 2.806 | 0.014 |
| LYM & CD3+ | 0.674 | 3.409 | 0.004 |
| CD3+ & CD8+ | 0.682 | 3.493 | 0.004 |

**Figure S1:**
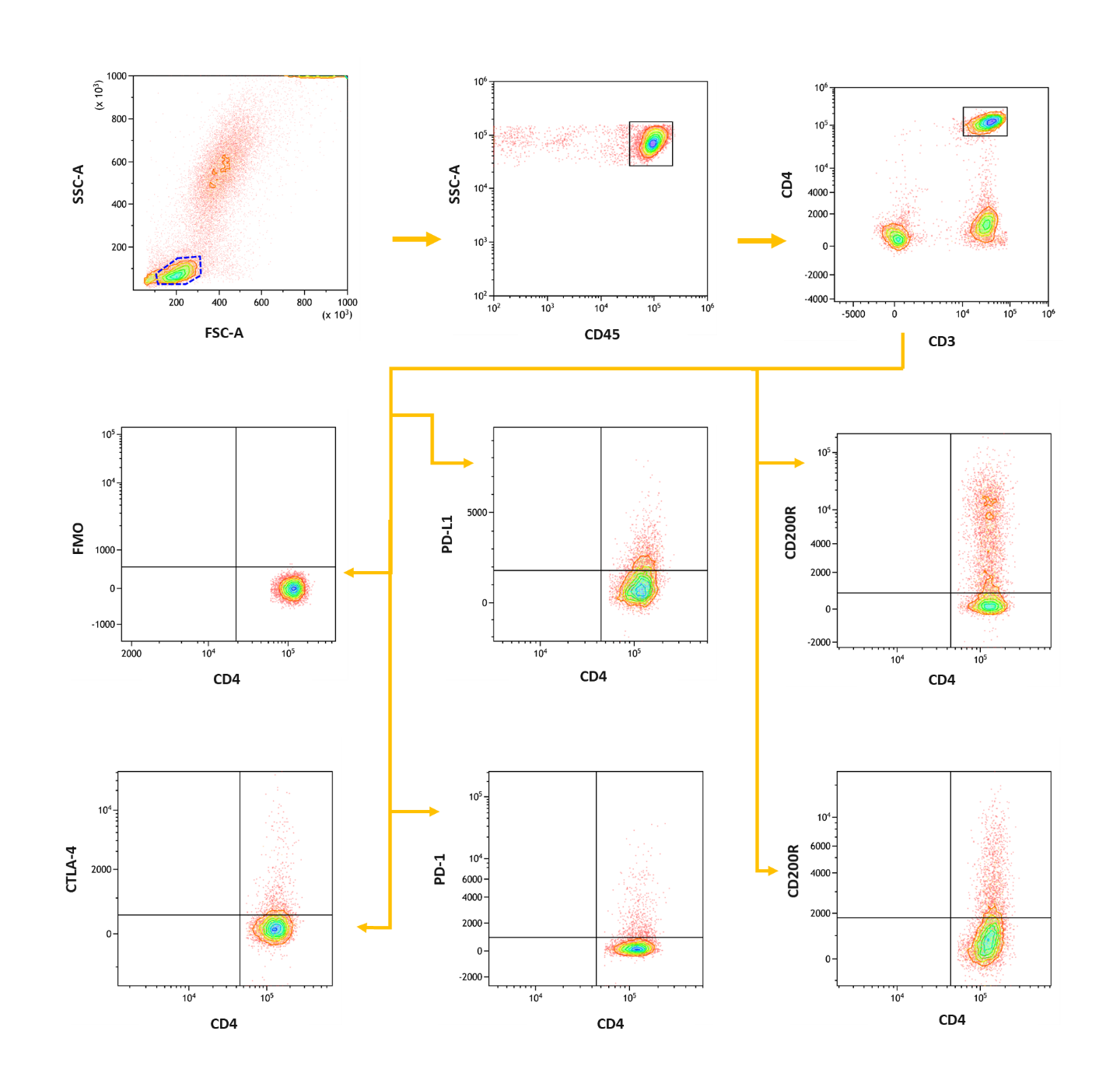
 An example cytometric analysis path for CD4+/CD3+ cells along with the assessment of the CD200+, CD200R+, PD-1+, PD-L1+, CTLA-4+ population. Analysis using FMO control. The remaining analyses for the CD8+/CD3+ and CD19+/CD3- subpopulations were performed similarly.
